# Identical effects of VEGF and serum-deprivation on phenotype and function of adipose-derived stromal cells from healthy donors and patients with ischemic heart disease

**DOI:** 10.1186/1479-5876-11-219

**Published:** 2013-09-18

**Authors:** Bjarke Follin, Josefine Tratwal, Mandana Haack-Sørensen, Jens Jørgen Elberg, Jens Kastrup, Annette Ekblond

**Affiliations:** 1 Cardiology Stem Cell Center, The Heart Center, Rigshospitalet, University Hospital Copenhagen, Copenhagen, Denmark; 2 Department of Plastic Surgery, Rigshospitalet, University Hospital Copenhagen, Copenhagen, Denmark

**Keywords:** Angiogenesis, Adipose-derived stromal cells, VEGF

## Abstract

**Background:**

Adipose-derived stromal cells (ASCs) stimulated with vascular endothelial growth factor (VEGF) and serum-deprived, are applied in the first in-man double-blind placebo-controlled MyStromalCell Trial, as a novel therapeutic option for treatment of ischemic heart disease (IHD). This *in vitro* study explored the effect of VEGF and serum deprivation on endothelial differentiation capacity of ASCs from healthy donors and IHD patients.

**Methods:**

ASCs stimulated with rhVEGF_A165_ in serum-deprived medium for one to three weeks were compared with ASCs in serum-deprived (2% fetal bovine serum) or complete medium (10% fetal bovine serum). Expression of VEGF receptors, endothelial and stem cell markers was measured using qPCR, flow cytometry and immunocytochemistry. *In vitro* tube formation and proliferation was also measured.

**Results:**

ASCs from VEGF-stimulated and serum-deprived medium significantly increased transcription of transcription factor *FOXF1*, endothelial marker *vWF* and receptor *VEGFR1* compared with ASCs from complete medium. ASCs maintained stem cell characteristics in all conditions. Tube formation of ASCs occurred in VEGF-stimulated and serum-deprived medium. The only difference between healthy and patient ASCs was a variation in proliferation rate.

**Conclusions:**

ASCs from IHD patients and healthy donors proved equally inclined to differentiate in endothelial direction by serum-deprivation, however with no visible additive effect of VEGF stimulation. The treatment did not result in complete endothelial differentiation, but priming towards endothelial lineage.

## Background

Ischemic heart disease (IHD) is the leading cause of death in industrialized countries and the incidence is continuously increasing due to the ageing population and ongoing obesity pandemic [1,2]. Despite improved medical treatment and interventional therapy in acute phases of the disease, a large number of patients still develop chronic heart disease or heart failure. It is of great interest to devise novel treatment options for those IHD patients who do not respond sufficiently to conventional care. One relatively new approach is the use of stem cell therapy with the potential to support regeneration of the damaged myocardium [3,4].

Mesenchymal stromal cells (MSCs) are unspecialized cells characterized by their ability to differentiate into various tissue-specific cells and to preserve the potential for both differentiation and self-renewal through multiple cell divisions [5]. They have been shown to possess regenerative abilities including differentiation and incorporation into regenerating tissue, immunomodulation, inhibition of apoptosis and scarring, stimulation of progenitor cells, and contribution to angiogenesis [6,7]. MSCs have been identified in various tissues in the human body including both adipose-derived stromal cells (ASCs/ADSCs) and bone marrow-derived stromal cells (BMSCs). BMSCs are currently the most thoroughly investigated, however, ASCs possess comparative abilities, and are harvested with minimally invasive liposuction with a higher yield of stem cells per harvest, and proliferate faster during *ex vivo* expansion. This makes them a more preferable source of stem cells for regenerative therapies [8-12].

A prerequisite for tissue regeneration in the ischemic heart is the reestablishment of blood supply to the infarct area. Therefore the effect of stem cell therapy is bound to be promoted by the vasculogenic or angiogenic ability of stem cells, and possibly so by their endothelial differentiation potential [13]. In order to increase the effect of stem cell treatment, it could prove beneficial to precondition the stem cells, in order to enhance these abilities.

It has been shown that BMSCs can differentiate towards an endothelial lineage by stimulation with vascular endothelial growth factor (VEGF), traditionally in combination with serum deprivation to suppress proliferation [14-17]. Our group has conducted a clinical study using BMSCs preconditioned for one week with rhVEGF-A_165_, the predominant human VEGF isoform, to stimulate endothelial differentiation of BMSCs before injection into IHD patients (NCT ID: NCT00644410) [18]. This study rendered the procedure feasible and safe [19,20]. As recently submitted by our group, a three-year follow-up found that patients treated with VEGF-stimulated BMSCs had increased exercise capacity and improvements in clinical symptoms compared to pre-treatment.

There are reports that ASCs can also differentiate into endothelium in vitro and in animal ischemia models [21,22]. As a consequence of the results from the previous BMSC trial and pre-clinical evidence for the beneficial use of ASCs, the randomized double-blind placebo-controlled MyStromalCell Trial was initiated (NCT ID: NCT01449032). MyStromalCell Trial is the first in-man clinical trial that uses culture-expanded ASC stimulated with rhVEGF-A_165_ a week prior to cell therapy treatment in patients with chronic myocardial ischemia and refractory angina [23].

Most previous and ongoing trials which have yielded promising results, apply autologous stem cells from patients [24]. However, the potential effect of age and disease on functional activity of the autologous stem cell pool is debated, and conflicting results have been published [25-28]. Our group found no difference in proliferation or endothelial differentiation between BMSCs from cardiac patients and healthy donors [29]. Human ASCs have been found to have decreased population doublings and markers of senescence with donor age [26]. However, a recent study found that ASCs from aged donors exhibited proliferation rates and osteogenic differentiation comparable to controls [28]. The only study investigating the abilities of ASCs with regard to endothelial differentiation from donors with heart disease, showed that it was feasible despite the disease, but no comparison with healthy controls was performed [30]. Therefore there is no exact knowledge about the potential differences that might exist between ASCs from patients with heart disease and healthy donors. Furthermore, no studies have investigated the effect of VEGF treatment on ASCs and the potential differences in this effect between ASCs from IHD patients and healthy donors.

The present *in vitro* study investigated the effect of the clinically applied VEGF pre-treatment of ASCs from IHD patients and healthy donors. Differentiation of ASCs towards endothelium after one, two, and three weeks of VEGF stimulation and serum deprivation was evaluated by identifying the presence of lineage specific markers on transcriptional and protein level as well as functional *in vitro* angiogenesis.

## Methods

### Donors

ASCs from 7 IHD patients (all males, 58 to 76 years old, mean age 65.7) enrolled in the placebo group of the MyStromalCell Trial were used. All had coronary artery bypass grafting and hyperlipidemia. One out of seven had diabetes mellitus and two out of seven had hypertension. In addition, ASCs from 7 healthy donors (2 male and 5 female, 28 to 57 years old, mean age 41.5) were used. The protocol for the clinical study is in line with the declaration of Helsinki, and approved by the National Ethical Committee (H-3-2-2009-149) and the Danish Medicines Agency (2612–2867). The inclusion and exclusion criteria for the study can be found in the original paper [23]. The use of adipose tissue from healthy volunteers is approved by the National Ethical Committee protocol no. H-3-2009-119.

### Isolation of ASCs

Approximately 100 ml lipoaspirate was obtained from liposuctions of subcutaneous abdominal fat performed under local anesthesia. The lipoaspirate was washed twice with phosphate buffered saline (PBS) pH 7.4 (GIBCO, Life Technologies, UK) to remove residual blood. The adipose tissue was digested by incubation with Collagenase NB 4 (SERVA Electrophoresis GmbH, Germany) dissolved in HBSS (2 mM Ca^2+^, GIBCO, Life Technologies, UK) at 37°C for 45 min. under constant rotation. The collagenase was neutralized with complete medium (Dulbecco’s Modified Eagle Medium, low glucose 1 g/l supplemented with 25 mM HEPES and L-Glutamin, PAA Labortatories, Austria), 10% Fetal Bovine Serum pharma grade (FBS, AA Labortatories, Austria), and 1% Penicillin/Streptomycin (P/S, GIBCO, Life Technologies, UK) and filtered through a 100 μl mesh (Cell Strainer, BD Bioscience, CA, US). The remaining cells were centrifuged at 1200 g for 10 min. at room temperature, re-suspended and counted using NucleoCounter® NC-100™ (Chemometec, Denmark) according to manufacturer’s instructions.

### Cell culture

Cells were seeded in T75-flasks (Thermo Fischer Scientific, MA, US) at a density of 4.5×10^6^ cells/flask in complete medium and incubated at standard conditions (37°C, 5% CO_2_ humidified air). After two days in culture, cells were washed to remove non-adhering cells. After approximately a week in culture, cells were detached with TrypLE® Select (GIBCO, Life Technologies, UK), resuspended, counted on NucleoCounter®, frozen 1×10^6^ cells/1 ml FBS with 5% DMSO (WAK-Chemie Medical GmbH, Germany) at −80°C in Nalgene® Mr.Frosty freezing container (Sigma-Aldrich, MO, US) and transferred to liquid nitrogen the following day for storage [31].

When initiating an experiment, ASC were rapidly thawed and seeded inT75-flask with media changed the following day. When the cultures reached 80% confluence, cells were passaged at 3×10^5^ cells/T75-flask for the experimental setup.

Human umbilical vein endothelial cells (HUVECs, Lonza, Switzerland) were cultured as control cells for endothelial markers. After thawing as described above, they were cultured at 3×10^5^ cells/T75-flask in endothelial growth medium-2 (EGM-2, Lonza, Switzerland) and incubated at 37°C and 5% CO_2_ with medium changed every three days. Experiments were performed in passage 3.

### Experimental VEGF protocol

ASCs were cultured in complete medium until 80% confluence, after which medium was changed to either complete medium, serum-deprived medium (DMEM added 2% FBS and 1% P/S) or serum-deprived medium added 50 ng/ml rhVEGF-A_165_ (rhVEGFA_165_, R&D Systems, MN, US) (VEGF stimulation medium). Media was renewed every three days and cells were cultured for one, two, or three weeks after which they were harvested for further processing.

### Nucleic acid extraction

At the end of stimulation, cells were detached with TrypLe and centrifuged at 300 g for 5 min. in RNase free tubes (BD Biosciences, CA, US). To the cell pellet 350 μl lysis buffer was added from the Qiagen RNeasy® Mini Kit (QIAGEN Hamburg GmbH, Germany) and a 1 ml syringe (B.Braun Melsungen AG, Germany) was used to lyse the cells with lysis buffer before applying the rest of the Qiagen protocol. Finally, total RNA was eluated with RNase-free water (5 Prime GmbH Hamburg, Germany) RNA purity was measured using a NanoDrop® 1000 Spectrophotometer (Thermo Scientific, MA, US), and the eluate was stored at −80°C. RNA purity was validated by absorbance ratios at 260 nm/280 nm and protein contamination at A260/230. RNA integrity was confirmed by RIN values >8 using RNA Nano Chips (Agilent Technologies, CA, US) and the Agilent 2100 Bioanalyzer with the instructions of the Agilent RNA 6000 Nano Kit.

### Reverse transcription

cDNA synthesis was prepared using AffinityScript (Stratagene, Agilent Technologies, CA, US) in a fast eight-tube strip (0.1 ml, MicroAmp™, Applied Biosystems®, Life Technologies, UK) on ice. The total reaction volume was 20 μl with 0.5 μg RNA, 10 μl cDNA synthesis master mix, 3 μl Oligo dT primer, 1 μl AffinityScript RT RNase block enzyme mixture, and RNAase-DNAse free water (5 Prime GmbH Hamburg, Germany) to 20 μl total volume. The reactions were performed with an initial stage of 25°C for 5 min., 42°C for 45 min., and 95°C for 5 min. (Veriti 96 well fast thermal cycler, Applied Biosystems®, Life Technologies, UK). Following synthesis, cDNA was stored in aliquots at −20°C.

### Quantiative real-time PCR

Brilliant II SYBR®Green QPCR master mix with low reference dye ROX (Agilent Technologies, CA, US) was used with a total reaction volume of 25 μl in 96-well optical reaction plates (Agilent Technologies, CA, US) with 5 μl of cDNA diluted 1:5 in 1× EDTA (QIAGEN Hamburg GmbH, Germany) and subsequently 1:5 in RNAase-DNAse free water (5 Prime GmbH Hamburg, Germany). The plate was sealed with optical plastic caps (Agilent Technologies, CA, US). qPCR was performed using Mx3000 (Stratagene, AH-diagnostics, Denmark) and the results were collected using Mx3000 version 4.0 software for Windows (Stratagene, AH-diagnostics, Denmark). The reaction was initiated by heating to 95°C for 10 min., followed by 40 cycles of elongation at 60°C for 1 min. and denaturation at 95°C for 30 sec.

### Target and reference genes

The genes of interest were MSC marker *CD105*, endothelial markers *Forkhead box protein F1 (FOXF1)*, *VEGF receptor 1* and *2 (VEGFR1* and *VEGFR2*), *von Willebrand factor* (*vWF)* and *CD31* with HUVECs as control (Table 1). To verify the efficiency of each run, a calibration curve of pooled cDNA diluted to fit a logarithmic curve was included on each plate. *Peptidylprolyl isomerase A (PPIA)* was selected as one of the most stable reference genes from a larger panel including *18S rRNA, beta-actin, elongation factor-1 alpha, glyceraldehyd 3-phosphate dehydrogenase, beta-glucoronidase, ribosomal protein L13a, TATA-binding protein,* and *tyrosine 3-monooxugenase/tryptophan 5-monooxygenase activation protein zeta peptide*. The choice was based on stability both in terms of donor variation and expression through the treatment. These comparisons were made by the GenEx software (Multid Analysis AB, Sweden). With the reference gene software subprograms geNorm and Normfinder we selected *PPIA* as the reference gene.

**Table 1:** Genes of interest and reference gene for qPCR

**Gene**	**Full name**	**GenBank accession number**	**Forward (F) and Reverse (R) sequence**	**Cellular function**
*CD105*	Cluster of differentiation 105/	NM_000118.2	F = 5′-AACACCATCGAGCCGGG-3′	TGF-β signalling, cytoskeletal organization
	Endoglin		R = 5′-GAACTCGGAGACGGATGGG-3′	
*VEGFR1 (FLT1)*	Vascular endothelial growth factor receptor 1 (FMS-like tyrosine kinase)	NM_002019.4	F = 5′-ATGCTGGATTGCTGGCACA-3′	Cell proliferation and differentiation
			R = 5′-TCAAACATGGAGGTGGCATT-3′	
*vWF*	von Willebrand Factor	NM_000552.3	F = 5′-CGGCTTGCACCATTCAGC-3′	Hemostasis, platelet adhesion
			R = 5′-CCATCCTGGAGCGTCTCATC-3′	
*FOXF1*	Forkhead box protein F1	NM_001451.2	F = 5′-CACTCCCTGGAGCAGCCGTATG-3′	Embryonic development, control of cell cycle
			R = 5′-AAGGCTTGATGTCTTGGTAGGT-3′	
*CD31 (PECAM-1)*	Cluster of differentiation 31	NM_000442.4	F = 5′-ACAGCCTTCAACAGAGCCAAC-3′	Leukocyte migration, angiogenesis
	Platelet cell adhesion molecule 1		R = 5′-GAAAGAATGACTCTGACTGTCAGTATT-3′	
*PPIA*	Peptidyl prolyl isomerase A	NM_021130.3	F = 5′-TCCTGGCATCTTGTCCATG-3′	Protein folding
			R = 5′-CCATCCAACCACTCAGTCTTG-3′	

Information for panel of chosen genes of interest and selected reference gene with full name, ncbi reference sequence, forward and reverse primer sequences, and short description of cellular function.

The fold changes in gene expression, normalized to the mean of *PPIA*, were calculated using the ΔΔCq method, with 2^ΔΔCq^ as the fold change [32].

### Gel electrophoresis

The presence of a single product from reference gene and genes of interest was verified with dissociation curves and by loading products from randomly selected qPCR for gel electrophoresis. A 3% NuSieve 3–1 agarose (Lonza, Switzerland) gel with 1× TAE buffer diluted from 50× TAE buffer (QIAGEN Siences, MD, US). To visualize PCR products, 10% non-toxic GelStar Nucleic Acid Gel Stain (Lonza, Switzerland) was added to the gel. Electrophoresis was performed in 1× TAE buffer. For every 8 μl PCR product, 2 μl Gelpilot 5× loading dye (QIAGEN Siences, MD, US) was added and loaded. A negative control was included to evaluate contamination of the product. Gelpilot 50 bp ladder and Gelpilot 1 kb ladder (both QIAGEN Siences, MD, US) were used. The gel was run between 70 and 100 volt in 45 – 60 min. and visualized under ultraviolet light (Uvidoc, Uvitec Cambridge, MA, US).

### Flow cytometry

ASCs from six subjects, (three healthy donors and three IHD patients), stimulated one to three weeks as described above, were detached using Accutase (PAA Laboratories, Austria), centrifuged, counted, and frozen. On the day of analysis, cells were thawed, washed in flow cytometry-PBS containing FACS-PBS (Hospital pharmacy, Copenhagen, Denmark), 1% EDTA (Hospital pharmacy, Copenhagen, Denmark), and 10% new born calf serum (GIBCO, Life Technologies, UK) and counted. Cells were re-suspended in flow cytometry-PBS, (1.5×10^5^ cells/100 μl) and incubated with or without initially titrated antibodies for 15 min. at room temperature. The antibodies were CD105-phycoerythrin (PE) (R&D System, UK), CD90-flourescein isothicyanate (FITC) (Beckman Coulter, Germany), CD73-PE (BD Bioscience, NJ, US), CD13-phycoerythrin and Texas Red (ECD) (Beckman Coulter, Germany), CD45-phycoerythrin-cyanin (PC7) (Beckman Coulter, Germany), CD34-allophycocyanin (APC) (Beckman Coulter, Germany), CD19-ECD (Beckman Coulter, Germany), CD14-PC7 (Beckman Coulter, Germany), HLA-DR-FITC (Beckman Coulter, Germany), VEGFR2-APC (R&D System, UK), Tie-2-PE (R&D System, UK), CD144-APC (R&D System, UK), CD31-PE (R&D System, UK). Isotypic controls used were IgG2aFITC, IgG1-ECD, IgG1-APC, MsIgG1-PC7 (all Beckman Coulter, Germany), and IgG1-PE (BD Bioscience, NJ, US) The cells were then centrifuged and re-suspended in PBS. Viability was determined by adding 1 μl of SYTOX blue 7 min. prior to analysis (SYTOX®, Invitrogen, Life Technologies, UK). HUVECs were used as positive controls for endothelial markers. Expression of endothelial markers and MSC characteristic surface markers as suggested by the ISCT [33] was measured by flow cytometry on a Navios flow cytometer (Beckman Coulter, Germany) using a six-color protocol. The protocol was developed with manual compensation, isotypic controls and Fluorescence Minus One. Dead cells and doublets were excluded from the final analysis. Data was analyzed using Navios software and Kaluza (Beckman Coulter, Germany).

### Immunocytochemistry

MSC markers as well as markers for endothelial differentiation were qualitatively assessed with immunofluorescence microscopy. Antibodies targeting stem cell markers were CD73 (1:50, polyclonal rabbit anti-human, Abcam, UK) and CD90 (1:100, monoclonal mouse anti-human, Stemgent, MA, US) while those targeting endothelial markers included vWF (ready to use, polyclonal rabbit anti-human, DAKO, Denmark), Tie-2 (1:200, polyclonal rabbit anti-human, Santa Cruz, TX, US), VEGFR2 (1:10, polyclonal goat anti-human, R&D System, UK), CD144 (1:1000, polyclonal rabbit anti-human, Nordic BioSite, Denmark), CD31 (1:100, monoclonal mouse anti-human, DAKO, Denmark), and FOXF1 (1:75, polyclonal rabbit anti-human, Abcam, UK).

ASCs from two healthy donors and two IHD patients were seeded at a density of 3×10^3^ cells/cm^2^ in four-chamber Permanox^TM^ chamberslides (Lab-Tek™ Chamber Slides, ThermoFisher Scientific, MA, US). Cells were treated with the three different media types for one, two and three weeks. Cultures were fixated with 4% Paraformaldehyde in PBS pH 7.4 (Hospital pharmacy, Denmark) for ten minutes. Unspecific binding was blocked with 2% Bovine Serum Albumin (BSA, Sigma Aldrich, MO, US). Primary antibodies were diluted according to initial titration in 1% BSA and cells were incubated with primary antibodies overnight at 4°C. Secondary antibodies were added for one hour at room temperature shielded from light. Secondary antibodies were Alexa Fluor® 555 goat anti-rabbit IgG, Alexa Fluor® 568 goat anti-rabbit IgG, Alexa Fluor® 568 goat anti-mouse IgG, Alexa Fluor® 555 goat anti-mouse IgG, Alexa Fluor® 568 donkey anti-goat (1:250, all from Invitrogen, Life Technologies, UK).

Primary antibodies were omitted in control wells to test secondary antibody specificity. Positive controls were done on HUVECs seeded 3×10^3^ cells/well in four-well chamber slides in EGM-2 medium (EGM-2 Bulletkit, Lonza, Switzerland) two days prior to fixation. After secondary antibody incubation, chambers were removed and the slides carefully washed and dried. ProLong Gold antifade reagent with DAPI (Molecular Probes®, Invitrogen, Life Technologies, UK ) was used to stain for nuclei and mount the slides with cover slips (Menzel-Glaser, Germany). Pictures were taken at 20× or 40× objective with an Olympus IX51 microscope and an Olympus DP71 digital camera and Olympus U-RFL-T fluorescence system. Image analysis was performed with ImageProPlus 7.0 (Media Cybernetics).

### *In vitro* angiogenesis assay

An ECMatrix® *in vitro* angiogenesis assay (*In Vitro* Angiogenesis Assay Kit, Millipore, MA, US) was performed to test the ability of ASCs to form tubules. ASCs from three healthy donors and three IHD patients were stimulated as described above. Cells were seeded in duplicate wells in 150 μL of their respective media in a 96-well plate (Nunc, Thermo Scientific, MA, US) with 1×10^4^ cells/well on ECMatrix® prepared according to manufacturer’s instructions. Following seeding of cells, 96-well plates were incubated at 37°C with 5% CO^2^ for 20 hours. Every four hours, three representative pictures were taken in each well using a phase contrast Olympus IX51 microscope equipped with an Olympus TL4 halogen light source and an Olympus DP71 digital camera. Image analysis was performed with ImageProPlus 7.0 (Media Cybernetics). For each picture, tube formation was quantified blinded, by manually counting number of polygons formed defined as areas enclosed by tube-like structures.

### Proliferation assay

Proliferation was assessed using Bromodeoxyuridine (BrdU) cell proliferation ELISA Kit (Roche®, Switzerland). ASCs from three healthy donors and three IHD patients were seeded in 96-well plates, 1.0×10^3^ cells/well in triplicate in complete medium. After adhering overnight, 10 μM BrdU was added to each well. Media and BrdU labeling was changed on day two, and additional BrdU was added to the media on day three. Fixing, labeling and washing were performed according to manufacturer’s instructions. After stopping the substrate reaction with 25 μL 1 M 2 N H_2_SO_4_ (vWR, PA, US) for one minute on a shaker (IKA VIBRAX VXR basic), the absorbance was measured with a Microplate Reader (BioRad, Model 680, CA, US) at wavelength 450 nm and reference wavelength 655 nm. BrdU incorporation was measured at hours 24, 48, 72, and 96.

### Statistics

For qPCR data normality was assessed graphically by comparison to normally distributed generated data in Microsoft® Office Excel® 2007, with all other data analysis being performed in IBM SPSS and SigmaPlot. For ECMatrix and flow cytometry data, distribution was determined using Kolmogorov-Smirnov and Shapiro-Wilk tests for normality. A mixed model repeated measures ANOVA was performed for qPCR data, *in vitro* angiogenesis data, flow cytometry data, and BrdU proliferation data with *p* < 0.05 considered significant.

## Results

### Transcription of endothelial markers *FOXF1*, *vWF*, and *VEGFR1*

**Figure 1:**
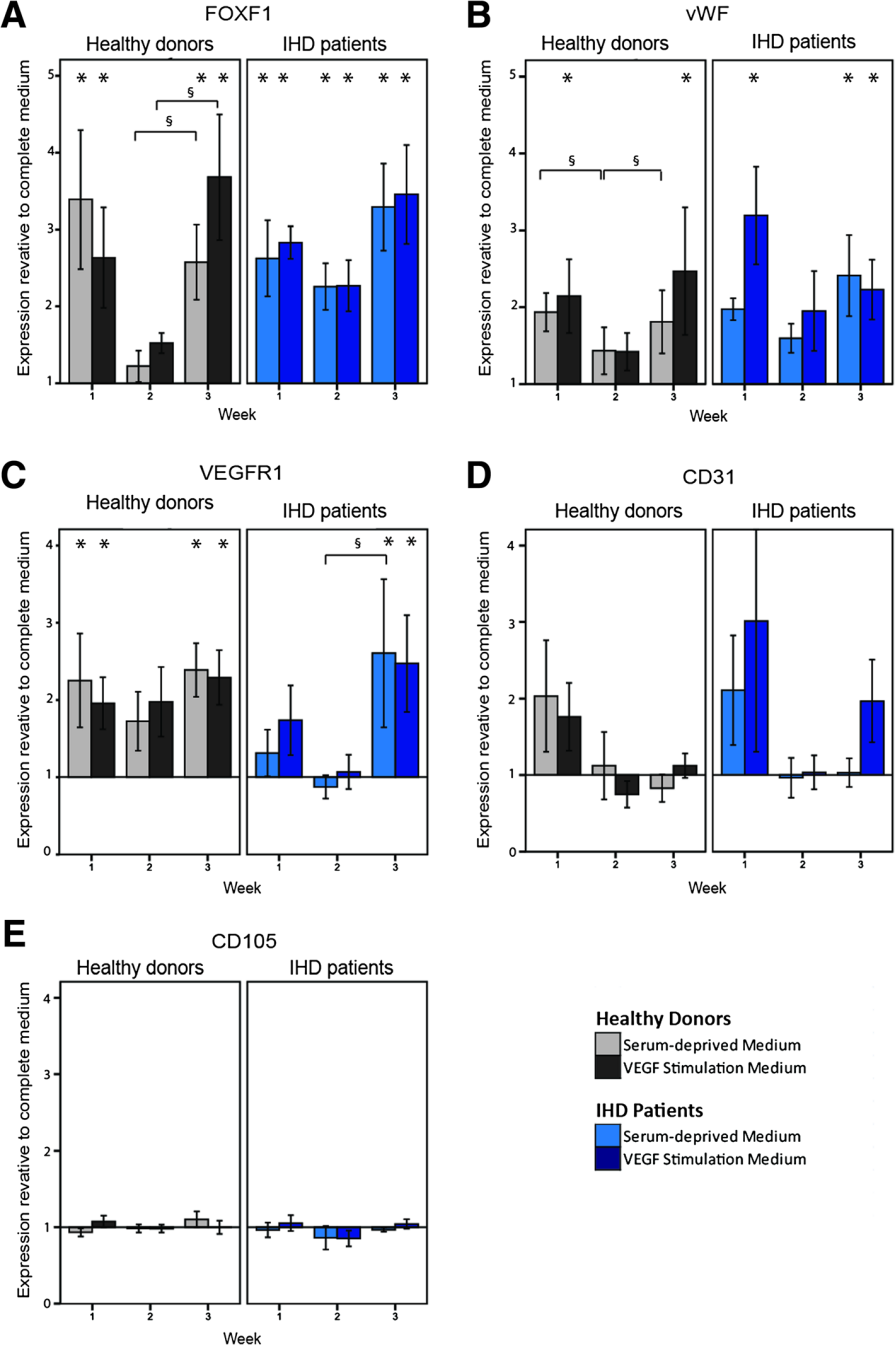
**Gene expression levels by quantitative real-time PCR analysis of ASCs.** Expression of FOXF1 **(A)**, vWF **(B)**, VEGFR1 **(C)**, CD31 **(D)**, and CD105 **(E)** in ASCs at passage two from both IHD patients (n = 7) and healthy donors (n = 7) in serum-deprived medium or VEGF stimulation medium. The expression of a gene is shown as fold change compared to expression of the same gene in ASCs from the same donor in complete medium, and has been calculated by the ∆∆Cq-method. Significant difference (*p* < 0.05) compared to complete medium is shown by * above the column, while a significant difference (*p* < 0.05) between the different weeks of culture is shown by §. ASCs; Adipose-derived stromal cells, IHD; ischemic heart disease, FOXF1; Forkhead box protein F1, vWF; von Willebrand factor, VEGFR1; vascular endothelial growth factor receptor 1, CD; Cluster of differentiation. Mean ± SEM.

We found that transcription of endothelial markers *FOXF1*, *vWF*, and *VEGFR1* were significantly up-regulated in serum-deprived and VEGF-stimulated ASCs compared to cells cultured in complete medium (Figure 1). There was no overall effect of donor health status on transcriptional activity, however we observed a tendency of lower expression of endothelial markers in week two which was not consistently significant. Relative to complete medium, expression levels of the early marker for endothelial differentiation *FOXF1* were significantly increased (p < 0.05) in ASCs when cultured in serum-deprived medium and VEGF stimulation medium for IHD patients for the three weeks tested. *FOXF1* was also significantly increased in ASCs from healthy donors at week one and three of culture (Figure 1A). The expression of endothelial marker *vWF* was significantly increased (p < 0.05) in all ASCs in VEGF stimulation medium after week one and three, and additionally for IHD patients in serum-deprived medium at week three (Figure 1B). The expression of *VEGFR1* was significantly increased (p < 0.05) for all ASCs in serum-deprived medium and VEGF stimulation medium at week three, and also after week one for healthy donor ASCs (Figure 1C). Transcription of stem cell marker *CD105* and endothelial marker *CD31* were not significantly affected by type of media, time in culture or donor health status , though we observed an insignificant trend of CD31 increased only in week one (Figure 1D and E). *VEGFR2* was very low expressed, with large variance between technical replicates and was therefore excluded. HUVECs were positive for endothelial markers. The Cq values for VEGF stimulated ASCs and HUVECs, respectively, were 31.0 ± 1.0 and 23.1 for *FOXF1*, 30.4 ± 1.3 and 26.2 for *vWF*, 30.8 ± 1.4 and 27.1 for *VEGFR1*, 21.0 ± 1.1 and 19.4 for *CD105*, and 33.0 ± 1.6 and 21.6 for *CD31*.

### Persistent stem cell characteristics on flow cytometry

**Figure 2:**
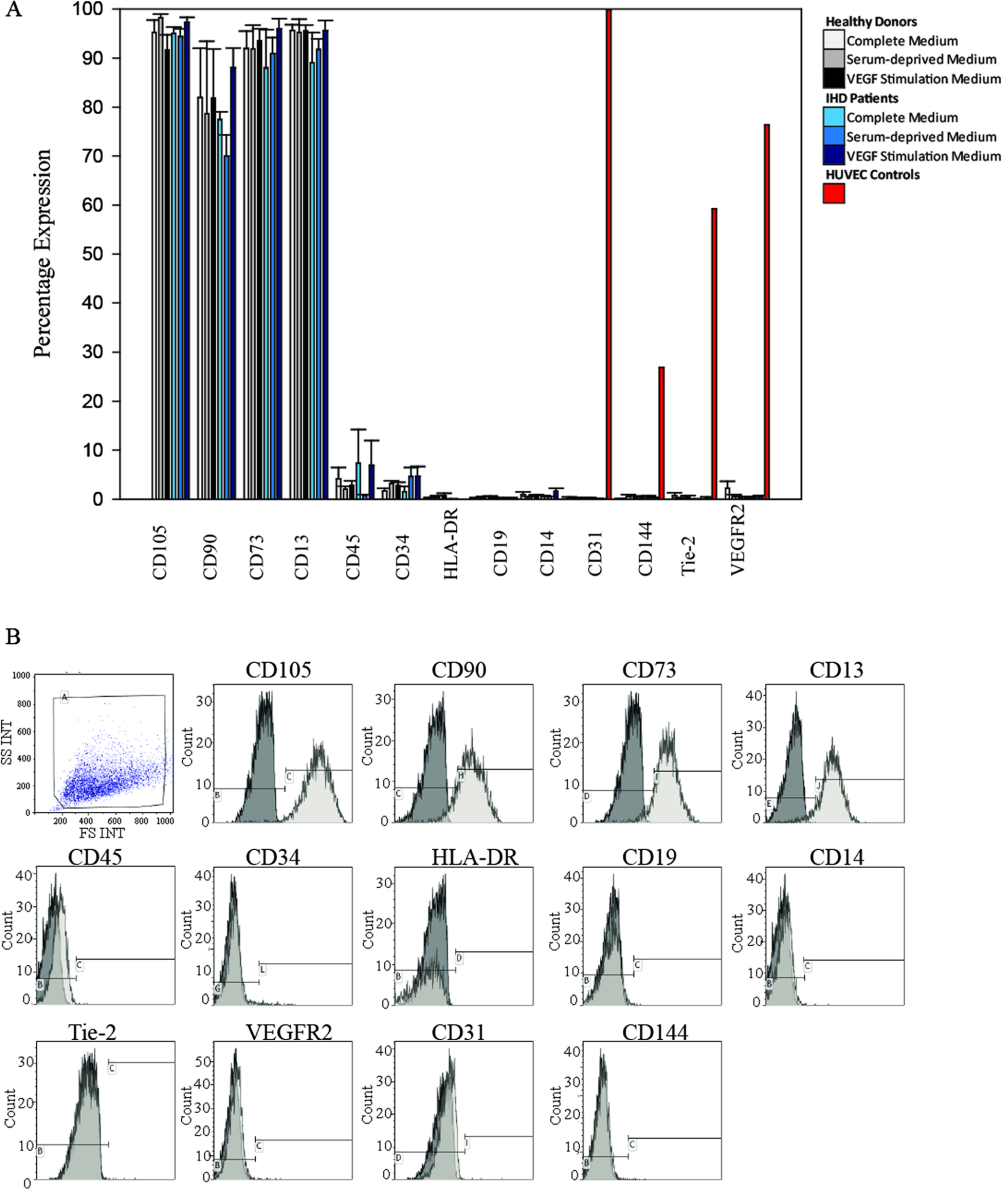
**Detection of ASC surface marker expression by flow cytometry.** Mean expression percentage of stem cells markers CD105, CD90, CD73, and CD13, immune stimulation marker HLA-DR, leukocyte marker CD45, endothelial progenitor marker CD34, B-cell marker CD19, monocyte marker CD14, and endothelial markers CD31, CD144, Tie-2, and VEGFR2 **(A)**. Data is from the first week of stimulation for ASCs in passage three. A representative scatterplot and histograms from one healthy donor **(B)** for positive (white) and non-positive (grey) populations. The population shown on the scatterplot consists only of viable cells, while the remaining dead cells are gated out with the use of SYTOX dead cell stain. Isotypic controls were used during implementation and proved no unspecific staining. ASCs; Adipose-derived stromal cells, IHD; Ischemic heart disease, CD; cluster of differentiation, HUVEC; human umbilical vein endothelial cell, HLA-DR; Human leukocyte antigen-DR, Tie-2; TEK tyrosine kinase endothelial-2, VEGFR2; vascular endothelial growth factor receptor 2. N = 3 for ASCs and n = 1 for HUVECs. Mean ± SEM.

We found that MSC characteristic markers as defined by ISCT, were unaffected by VEGF treatment, donor health status or time in culture. Therefore, Figure 2A showing data from donors and patients is representative for all weeks. Likewise, Figure 2B depicting expression from week one in ASCs from a healthy donor is representative for all weeks and donor health status. Positive MSC CD105, CD73, and CD13 were all expressed at a level around 95%, while CD90 expression was around 80%. Expression of the markers HLA-DR, CD19, and CD14 was below 2%, while the expression level of CD45 and CD34 was between 2% and 8%. Endothelial markers, CD31, VEGFR2, CD144 and Tie2 were below 2% in expression levels, while these were at the levels of 99%, 76%, 27%, and 59% for HUVEC controls, respectively. The ASC population is shown on a scatter plot together with representative overlay plots for each marker in Figure 2B.

### Visualization of endothelial and stem cell markers by immunocytochemistry

**Figure 3:**
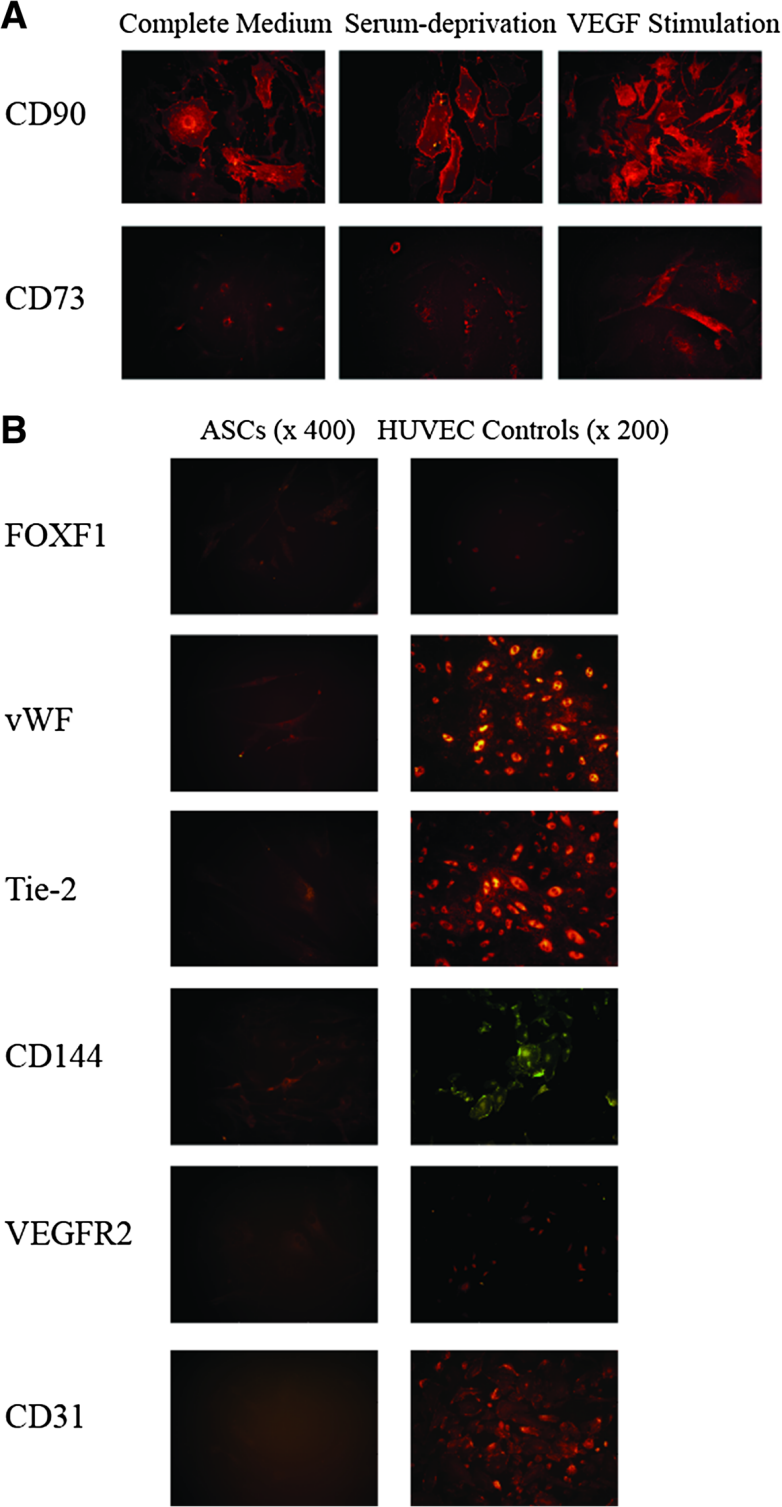
**Stem cell expression visualized by fluorescent immunocytochemistry.** Representative pictures from a healthy ASC donor for CD90 and CD73 visualized at × 40 for all media types **(A)**. Pictures of the sporadic positivity for some of the endothelial markers at × 40 against HUVEC controls at × 20 **(B)**. ASC; Adipose-derived stromal cell, CD; Cluster of differentiation, HUVEC; human umbilical vein endothelial cell.

Protein expression was visualized with immunocytochemistry by staining for endothelial markers FOXF1, VEGFR2, vWF, Tie-2, CD144, CD31, and MSC markers CD90 and CD73. Both of the MSC markers were present across all weeks, culture conditions and donors, shown with representative images from healthy donors week one in Figure 3A. All endothelial markers were identified on positive control cells (HUVEC). However, on ASCs we found only sporadic unsystematic staining of endothelial markers across all weeks, culture conditions and donors. Figure 3B presents the positive sporadic expressions on ASCs together with HUVEC controls.

### Serum-deprivation has a positive effect on *in vitro* angiogenesis

**Figure 4:**
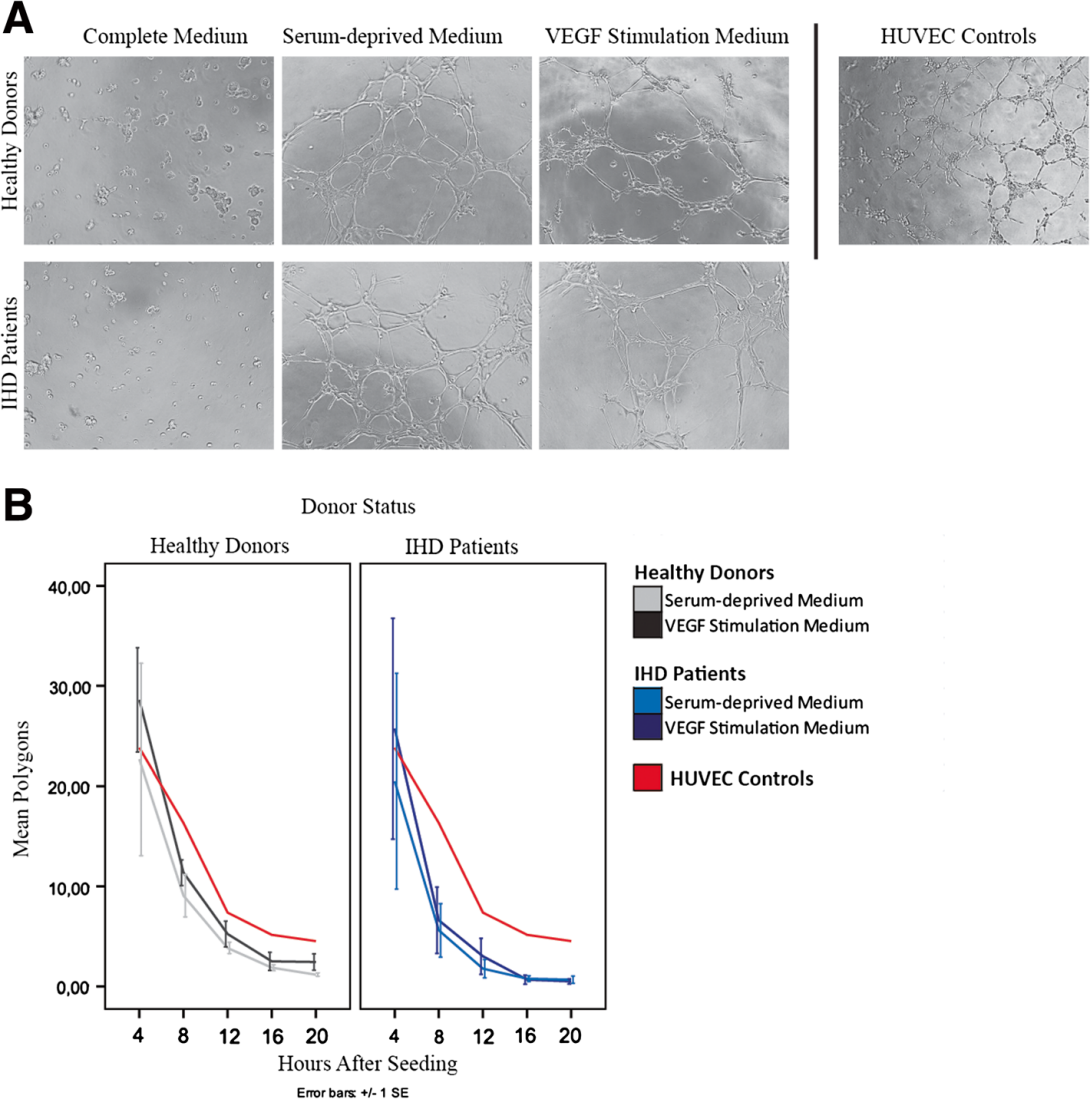
**Tube formation of ASCs after 4 hours on ECMatrix.** Representative pictures of ASCs from IHD patients and healthy donors in complete medium, serum-deprived medium, and VEGF stimulation medium **(A)**. Timeline of generated number of polygons after one week of stimulation, representative for all weeks, with HUVEC reference timeline **(B)**. N = 3 for ASCs and n = 1 for HUVECs. ASC; Adipose-derived stromal cells, IHD; ischemic heart disease, HUVEC; human umbilical vein endothelial cell.

A clear difference in tube formation was observed for ASCs in serum-deprived medium and VEGF stimulation medium compared to complete medium which contributed with little to no tubulogenesis (Figure 4A). ASCs in complete medium gathered in spheres rather than tubes, and polygons were seldom observed. Both patient and healthy donor ASCs showed the greatest tube formation after four hours. Calculated mean numbers of polygons presented no difference in tube formation between ASCs from serum-deprived medium and VEGF stimulation medium from either IHD patients or healthy donors (Figure 4B). Therefore serum deprivation seems to be the decisive factor with regard to tube formation for ASCs on ECMatrix®. ASCs were able to form the same amounts of polygons as HUVEC controls, and though the HUVEC polygons seemed slightly more stable at eight hours, their tube formation followed the same pattern as the ASCs.

### Decreased proliferation rate for ASCs from IHD patients

**Figure 5:**
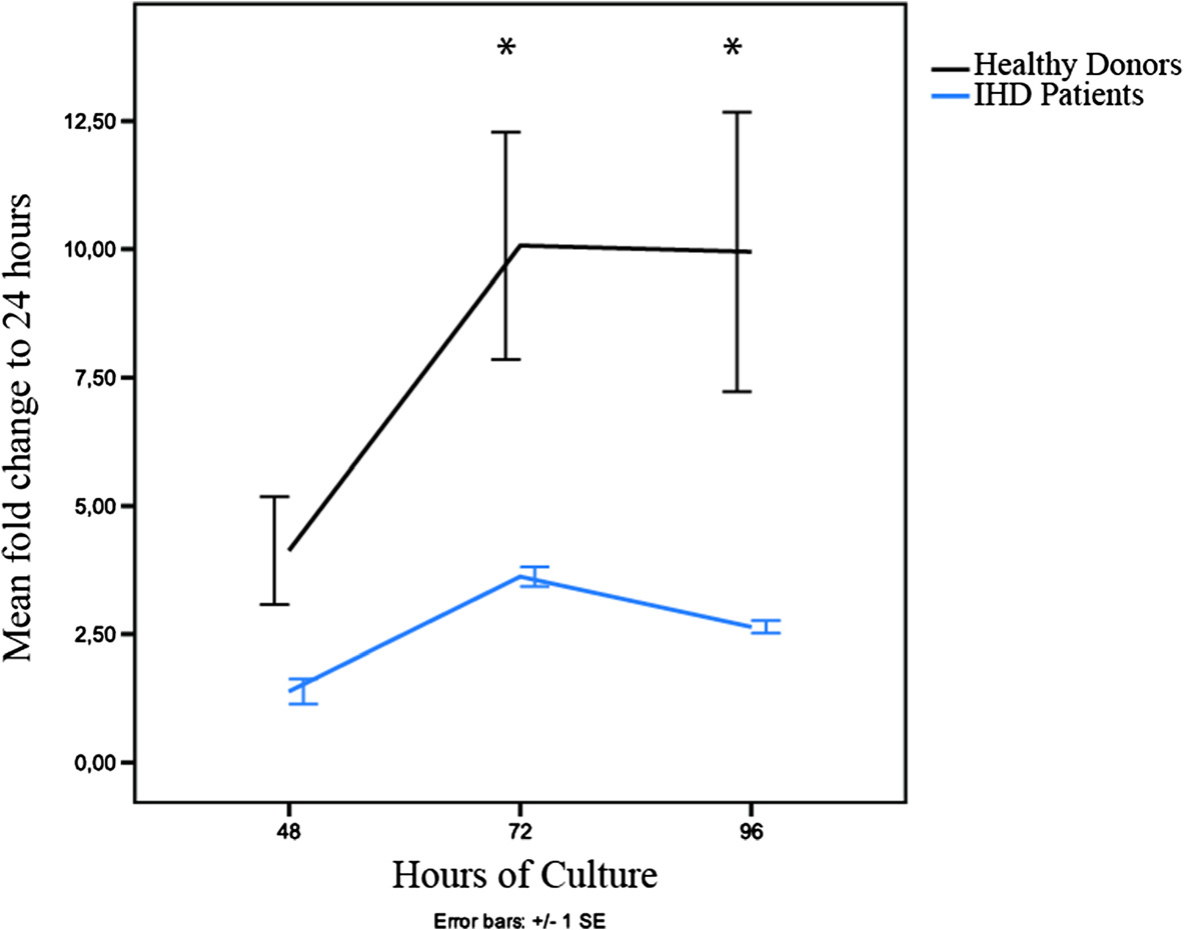
**ASC proliferation over four days.** Comparison between proliferation of ASCs from IHD patients (yellow) (n = 3) and healthy donors (green) (n = 3) using colorimetric BrdU proliferation assay measured by absorbance at 450 nm, reference 655 nm, at 24, 48, 72, and 96 hours, and normalized to 24 hours. ASCs; adipose-derived stromal cells, BrdU: Bromodeoxyuridine, IHD; ischemic heart disease Mean +/- SEM.

The proliferation rate was measured with BrdU incorporation over 96 hours for ASCs from three IHD patients and three healthy donors in complete medium. IHD patient ASCs exhibited significantly (*p* < 0.05) lower proliferation after 72 and 96 hours, measured as fold change from 24 hours (Figure 5). The slopes of the curves suggest a generally increased population doubling for ASCs from healthy donors resulting in the significant difference at 72 hours. This difference remained at 96 hours, though the proliferation of ASCs from both groups had stopped. The results only reflect proliferation, while the viability was not measured.

## Discussion

In the clinical MyStromalCell Trial the aim is to revascularize ischemic myocardial tissue by injection with ASCs that have been pre-treated with VEGF *in vitro* for one week, in order to improve *in vivo* angiogenic or vasculogenic function. We investigated the effect of such *in vitro* VEGF treatment in combination with serum deprivation for up to three weeks of ASCs from IHD patients and healthy donors with regard to differentiation towards an endothelial lineage. MSC and endothelial markers were tested for gene expression and protein levels, while functional tube formation and proliferation assays contributed to further characterization. The present *in vitro* study shows that pre-treatment of ASCs can guide these cells towards endothelial behavior, with serum deprivation and not VEGF as the decisive factor.

With regard to upregulation of markers *FOXF1* and *VEGFR1,* serum deprivation proved equally efficient alone and in combination with VEGF. Serum deprivation did not produce the same effect as VEGF treatment, in terms of *vWF* expression after one and three weeks of stimulation for healthy donors. This could be attributed to a genuine effect of VEGF. However, since the difference between the VEGF treatment and the serum-deprivation is not significant and as this is the only marker and time point which differs between the two treatments, we cannot exclude this as a figment of donor variation. We included the transcription factor *FOXF1* as an early marker of endothelial differentiation, because its activation in the mesoderm leads to *VEGFR2* expression and activation of vascular tube formation [34]. The precise functions of *FOXF1* have not yet been elucidated, but it has been implicated in endothelial-mesenchymal transition [35-37]. The co-expression of *FOXF1*, *vWF*, and *VEGFR1* in our experiment, indicates that it is implicated in early stage differentiation towards endothelial lineage, possibly through cell cycle control needed for differentiation to occur [36].

We failed to identify expression of *VEGFR2* on ASCs in all tested circumstances. These data are in compliance with *Ball et al.* who found neither *VEGFR2* nor *VEGFR1* on BMSCs [38]. *VEGFR2* has a function in cell migration and proliferation with regard to angiogenesis whereas the specific functional effect of *VEGFR1* is less well known [39,40]. It has been suggested that *VEGFR1* is a negative regulator of VEGF-A/*VEGFR2* signaling and can regulate signaling through *VEGFR2* by binding and sequestering VEGF-A, to which *VEGFR1* has a higher affinity than *VEGFR2*[39-43]. It has also been proposed that *VEGFR1* is a positive regulator of sprout formation and migration, possibly by negative control of the amount of VEGF-A sensed by the cells, which could explain our observations of *VEGFR1* increase and enhanced sprouting in serum-deprived conditions [40,44].

We found a general tendency for lower expression of all endothelial markers after the second week in culture. Inadequate stimulation may cause a transient feedback mechanism, resulting in the observed decrease in endothelial gene expression in week two. The lack of a continuous increase in expression during weeks in culture supports the idea that serum-deprivation only prepares the ASCs for differentiation but is insufficient to fully differentiate the ASCs toward endothelium, and even prolonged stimulation does not compensate for the need of multiple stimuli to carry the process through.

Immunocytochemistry with endothelial markers resulted in only sporadic staining independent of stimulation, time in culture and donors, even with the markers clearly up regulated at the transcriptional level. Acknowledging the heterogeneity of the population, however homogenous it appears according to the ISCT criteria, differences found in transcriptional activity might reflect changes in only a small subset of ASCs and not the population in general [45,46]. However, the sporadic staining was also observed in ASC cultured in complete medium, and did not correlate with the qPCR results. A more general subpopulation could be caused by endothelial cell contamination, which would explain the sporadic staining. An equally plausible explanation is that translation of given endothelial markers and receptors is never completed, but mRNA modulated due to lack of sequential stimuli in this in vitro scenario.

The fact that the ASCs readily form tubules when seeded on ECMatrix® in serum-deprived medium with and without VEGF demonstrates that they are capable of behaving like endothelial cells and on an initial level of tube formation similar to HUVEC controls, but need other stimuli, such as the ECMatrix® substrate, in addition to serum-deprivation and/or VEGF stimulation to commit to differentiation. Since there was no tubule formation in complete medium and ASCs did not spontaneously form tubules in the regular culture dish during serum-deprivation, neither ECMatrix® nor serum-deprivation is independently sufficient to stimulate ASCs to form tubules, emphasizing the need for multiple stimuli. This is supported by publications from several other groups which have successfully differentiated ASCs toward endothelial lineage with VEGF treatment but always in combination with additional factors. [30,47]. A multitude of parameters including endothelial cell growth supplements, shear force, three-dimensional culture and supporting matrices have also been found essential when addressing full endothelial differentiation of ASCs and BMSCs respectively [48-50].

The proliferation curve obtained from the BrdU incorporation assay suggests a slower population doubling of ASCs from IHD patients compared to those from healthy donors, which is similar to what others have found [27,51]. However, since there were no observed differences in the other measured parameters of the study, we conclude that the difference between ASCs from healthy donors and IHD patients is minimal, and only significant for proliferation.

ASCs retained their MSC characteristics defined by ISCT criteria under all tested circumstances as shown by flow cytometry. This was supported by the unaffected translation of *CD105* as well as protein expression of CD90 and CD73 identified with immunocytochemistry. The expression percentages of the MSC markers closely resemble the percentages presented in the ISCT criteria [33,52]. We obtained a larger than expected percentage of CD45+ cells. This was found to be related to samples with low viability by observing a high percentage of SYTOX® uptake in the same samples. The close correlation between poor viability and CD45 positivity could be due to unspecific CD45 labeling of dead and thereby permeable cells. Dead cells did not interfere with results for other markers. Taking this into consideration, patient and donor ASCs retain their MSC characteristic markers defined by ISCT stem cell definition standards, during culture with the different types of media including treatment with VEGF.

The observed impact of serum-deprivation is consistent with a recent study performed on BMSCs, where the cells that were serum-deprived over a longer period of time exhibited an endothelial phenotype, with an upregulation of angiogenic markers [53]. The limited response in upregulation of classical endothelial markers after VEGF stimulation and serum deprivation of the ASCs was not expected, since *Haack-Sorensen et al.* successfully induced the expression of *VEGFR2*, *vWF* and *CD31* mRNA and protein in BMSCs after a week in culture under identical circumstances [15]*.* This calls attention to the fact that BMSC and ASCs are not analogous, though this remains to be investigated in a side-by-side comparative study. However, endothelial differentiation of ASCs seems plausible provided more factors are present. The equal responses of the ASCs from healthy donors and IHD patients do not favor one over the other in terms of future allogenic or autologeous transplants.

The study has certain limitations. The controls for endothelial phenotype were HUVECs, while a more suitable control for this study could have been fully differentiated endothelial precursor cells derived from ASCs, using a more effective protocol [49]. In addition, we did not actively deplete endothelial cell contamination during ASC isolation. Though we rely on a previously established protocol, which has shown elimination of endothelial markers during culture, such endothelial cells could be speculated to perform endothelial-to-mesenchymal transition which could be reversible [15,54]. In terms of endothelial marker expression this could explain the subtle response on transcription and the sporadic appearance of markers with immunocytochemistry, while the percentage of cells remained below the 2% detection threshold for flowcytometry. The donor groups were not matched with age and sexes, which could give rise to selection bias. Differences in proliferative properties due to age have been reported, but the authors could not find data on gender differences in ASCs abilities [26]. The effect of co-morbidities was not investigated, since the results obtained were very similar in the IHD patient group. Finally, there has in recent years been a shift in paradigm in the field of stem cell research, towards the ASC secretome as the main contributor in stem cell therapy [55-57]. Enhancing pro angiogenic paracrine properties of ASCs has been achieved by different approaches [58,59]. In fact, a recent study by *Yan et al.*, found that VEGF-stimulated ASCs secreted increased amounts of VEGF themselves [59]. Thus, the cell-free conditioned medium from present study should be addressed in future studies with regards to potential differences in the secreted amounts of angiogenic growth factors.

## Conclusions

Our study found evidence of ASC differentiation in progress towards an endothelial lineage upon serum-deprivation, but we found no additional visible effect of VEGF stimulation or prolonged time in culture. The stimulatory effect of serum deprivation was subtle, with significant increase of only the earliest markers of endothelial differentiation on mRNA level. Serum-deprived ASCs however readily form tube-like structures on ECMatrix® even though traditional stem cell markers are maintained under all conditions. We did not find any difference between ASCs from healthy donors and IHD patients with regard to their *in vitro* differentiation capacity or retention of stem cell characteristics, but observed a slight difference in proliferative abilities.

Our study was conducted in support of the ongoing clinical MyStromalCell Trial. Though final endothelial differentiation of ASCs is not obtained during *in vitro* cultivation, we find it plausible that the demonstrated *in vitro* priming facilitates angiogenesis upon *in vivo* delivery into an environment with the necessary multi-factorial and three-dimensional setting. In terms of endothelial priming for future studies, it is not advantageous to use VEGF as an independent supplement, since it is expensive and no more effective than serum-deprivation.

### Consent

For the publication of this report, together with any accompanying images, written informed consent was obtained from the patients.

## Abbreviations

ASC/ADSC: Adipose-derived stromal cell; BMSC: Bone-marrow derived stromal cell; FOXF1: Forkhead box protein F1; HUVEC: Human umbilical vascular endothelial cell; IHD: Ischemic heart disease; ISCT: International society for cellular therapy; MSC: Mesenchymal stromal cell; PPIA: Peptidylpropyl isomerase A; qPCR: Reverse transcriptase quantitative real-time polymerase chain reaction; Tie-2: TEK tyrosine kinase endothelial 2; VE-cadherin: Vascular endothelial cadherin; VEGF: Vascular endothelial growth factor; VEGFR1: VEGF receptor 1; VEGFR2: VEGF receptor 2; vWF: von Willebrand Factor.

## Competing interests

The authors declare that they have no competing interests.

## Authors’ contributions

JT and BFL carried out the experiments, participated in the design of the study, performed the statistical analysis and drafted the manuscript. MHS participated in the design of the study, analysis and interpretation of data. JJE made substantial contributions in terms of liposuction procedures. JK initiated the study and revised the manuscript critically. AE conceived of the study, contributed in design and coordination and revised the manuscript critically. All authors read and approved the final manuscript.
